# Comparison of the Efficacy of Gabapentin Versus Levodopa-C for the Treatment of Restless Legs Syndrome in End-Stage Renal Disease on Hemodialysis Patients

**DOI:** 10.7759/cureus.12034

**Published:** 2020-12-11

**Authors:** Muhammad Ali, Hina Iram, Fahad Nasim, Shafique A Solangi, Abdul Manan Junejo, Noor Un Nisa, Sagheer Ahmed Solangi

**Affiliations:** 1 Department of Nephrology, Fazaia Ruth Pfau Medical College, Karachi, PAK; 2 Department of Nephrology, Jinnah Postgraduate Medical Centre, Karachi, PAK; 3 Department of Nephrology, Liaquat National Hospital, Karachi, PAK; 4 Physiology, Jinnah Sindh Medical University, Karachi, PAK; 5 Department of Internal Medicine, Jinnah Postgraduate Medical Centre, Karachi, PAK

**Keywords:** restless leg, gabapentin, pittsburgh, levodopa, sleep

## Abstract

Objective: We aimed to compare the efficacy of gabapentin and levodopa-c for the symptoms of restless leg syndrome in patients of end-stage renal disease (ESRD) undergoing maintenance hemodialysis therapy.

Methods: In this observational, cross-sectional study, patients of ESRD on hemodialysis with restless leg syndrome were included after assessment of symptoms and quality of sleep before the treatment by completing two questionnaires: the International Restless Leg Syndrome Study Group (IRLSSG) questionnaire and the Pittsburgh Sleep Quality Index (PSQI) scale. They were randomly divided into two groups. One group was prescribed levodopa-c (110 mg) as a single dose two hours before bedtime for four weeks. The other group was given gabapentin (200 mg) after each hemodialysis session for four weeks. After the treatment, the patients completed the two questionnaires again: the IRLSSG questionnaire and the Pittsburgh Sleep Quality Index.

Results: In our study, men were 14 (53.8%), and women were 12 (46.2%). Gabapentin was given to 14 (53.8%) patients, and 12 (46.2%) patients were prescribed levodopa-c. In the levodopa group, the average baseline IRLSS was 24.333 ± 7.936), and the mean baseline PSQI score was 13.583 ± 3.396. After treatment with levodopa for four weeks, the mean IRLSS was 8.666 ± 3.312, and the mean PSQI score was 4.666 ± 2.839; a P-value of 0.00001 was noted. While in the gabapentin group, the mean baseline IRLSS was 26.071 ± 7.936, and the mean baseline PSQI score was 14.857 ± 3.254. After treatment for four weeks with gabapentin, the mean IRLSS was 5.3571 ± 1.392, and the post-treatment average PSQI was 2.992 (SD: 0.916); a P-value of 0.00001 was noted.

Conclusion: Both levodopa and gabapentin effectively relieve symptoms of restless leg syndrome and improve the quality of sleep and life in ESRD patients undergoing hemodialysis.

## Introduction

The burden of kidney disease has increased in the past few decades. The prevalence of global kidney disease ranges from 11% to 13% [1]. In Pakistan, its prevalence is 14.3% and has affected 14.6 million people [2]. The majority of the end-stage renal disease (ESRD) patients undergoing hemodialysis have sleep disturbance that affects their quality of life; Insomnia, sleep-related breathing disorders, hypoventilation, central sleep apnea, and sleep-related movement disorders or restless leg syndrome is commonly found [3]. 

Restless leg syndrome (RLS) is a neurological disorder that manifests as an uncomfortable or unpleasant sensation in the lower limbs and a desire to move them constantly. Patients described this sensation as creeping, pulling, and throbbing feeling. The prevalence of restless leg syndrome is 5% to 15% in the general population, and the prevalence in patients undergoing hemodialysis therapy ranges from 8.8% to 83% [4]. Restless leg syndrome does not only affects the sleep pattern but also increases the patient's risk for cardiovascular diseases and increases the morbidity and mortality among ESRD patients [5].

In 2012, the International Restless Syndrome Study Group (IRLSSG) developed the diagnostic criteria for restless leg syndrome (RLS), which consists of five major points for its diagnosis (all must be met) [6]:

(a) An urge to move the legs, usually but not always, accompanied by or felt to be caused by uncomfortable and unpleasant sensations in the legs

(b) The urge to move the legs and any accompanying unpleasant sensations begin or worsen during periods of rest or inactivity such as lying down or sitting

(c) The urge to move the legs and any accompanying unpleasant sensations are partially or totally relieved by movement, such as walking or stretching, at least as long as the activity continues

(d) The urge to move the legs and any accompanying unpleasant sensations during rest or inactivity only occur or are worse in the evening or night than during the day

(e) The occurrence of the above features are not solely accounted for as symptoms primary to another medical or a behavioral condition (e.g., myalgia, venous stasis, leg edema, arthritis, leg cramps, positional discomfort, habitual foot tapping)

The pathophysiology of idiopathic RLS is poorly understood and multifactorial in ESRD patients undergoing hemodialysis. One of them is the dopaminergic system dysfunction [7]. Dopamine agonists are considered the first-line agents for RLS treatment and are widely used for its treatment [8]. But rebound, augmentation, and adverse effects of drugs hinder its use. Recently, another drug that is commonly used for neurological disorders is gabapentin, which is an analog of gamma-aminobutyric acid (GABA). It has shown significant improvement in the management of RLS symptoms, and it has also improved sleep time and quality of life. But the efficiency of this drug is not studied enough in hemodialysis patients.

The purpose of this study was to evaluate the efficacy of gabapentin and levodopa-c for the relieving of symptoms of RLS and improvement in the quality of sleep in ESRD patients on hemodialysis.

## Materials and methods

An observational, cross-sectional study was conducted at the Department of Nephrology, Jinnah Postgraduate Medical Centre, Karachi, Pakistan, from 1st June 2020 to 30th June 2020. All patients who were diagnosed as a case of restless leg syndrome after using predefined criteria [6] were enrolled in this study after taking written and informed consent. All the patients were randomly divided into two groups: group A and group B. Group A was prescribed levodopa-c (110 mg), single-dose two hours before bedtime, and group B was prescribed gabapentin (200 mg) after each hemodialysis session, for four weeks. Each patient completed two questionnaires: the International Restless Syndrome Study Group (IRLSSG) questionnaire and the Pittsburgh Sleep Quality Index (PSQI) scale. The international restless leg syndrome score (IRLSS) rating scale was proposed by the IRLSSG, which comprises 10 questions, and each question was graded from zero as none to four as very severe. The PSQI score was calculated by summing the scores. It ranges from 0-24 points. PSQI scores less than five indicate good sleep, and a score above five indicates poor sleep quality, whereby a higher score indicates worse sleep conditions. It has a diagnostic sensitivity of 100% and specificity of 93% [9]. Both the questionnaires were assessed before and after the treatment. 

Baseline characters of patients like age, gender, hemoglobin, serum calcium, phosphorous, albumin, iron, intact parathyroid hormone (iPTH), vitamin D level, frequency of hemodialysis, duration of hemodialysis, urea reduction ratio (URR), and single pool Kt/V were noted.

Data were analyzed with the Statistical Package for Social Sciences (SPSS) version 20 (IBM SPSS Statistics, Armonk, NY). Independent t-tests and the chi-squared tests were performed.

## Results

We studied 26 patients diagnosed with restless leg syndrome. The mean age was found to be 45.51 ± 13.29 years (Table 1).

**Table 1:** Biochemical characteristics of patients on hemodialysis Abbreviations. URR: urea reduction ratio; SpKt/V: single pool Kt/V where Kt/V shows dialysis adequacy by incorporating dialyzer clearance of urea (K), dialysis time (t), and volume of distribution of urea (V); iPTH: intact parathyroid hormone; Ses-week: sessions per week; S: serum

Variables	Mean ± Std. Dev
Age	45.51 ± 13.29
Dialysis Ses-week	2.606 ± 0.5126
Dialysis Duration/ Years	5.212 ± 1.004
URR	64.074 ± 4.404
SpKt/V	1.225 ± 0.115
Hb	9.27 ± 1.387
S. Vit-D	24.36 ± 13.03
S. iPTH	578.72 ± 583.15
S. Iron	91.50 ± 41.61
S. Calcium	8.11 ± 0.824
S. Albumin	3.33 ± 0.538
S. Creatinine	7.95 ± 2.50

Most patients (61.7%) were on four hours, thrice per week hemodialysis, and 37.2% were twice per week with an overall mean of 2.606 hours and a standard deviation of 0.5126 hours.

In our study, 53.2% of patients were on hemodialysis for more than five years. The mean urea reduction ratio was 64.074 mg/dl, and the single pool Kt/V was 1.225 (Table 1). In our study, the patient's mean hemoglobin level was 9.27 g/dl, mean iron 91.50 µg/dl, vitamin D deficiency was found with a mean value of 24.36 ng/dl. Of the 26 patients with restless leg syndrome, 14 (53.8%) were male, and 12 (46.2%) female. Gabapentin was given to 14 (53.8%) RLS patients, while levodopa was prescribed to 12 (46.2%) (Table 2).

**Table 2:** Gender and drug distribution Abbreviations. RLS: restless leg syndrome

Variables	Frequency
RLS Positive	26 (100%)
Male	14 (53.8%)
Female	12 (46.2%)
Group A (Levodopa-c)	12 (46.2%)
Group B (Gabapentin)	14 (53.8%)

In the levodopa group, the mean baseline IRLSS was 24.333 ± 5.928, and after treatment with levodopa for four weeks, the mean IRLSS was 8.666 ± 3.312 (P-value: 0.0001). Similarly, the mean baseline PSQI score was 13.583 ± 3.396. After treatment with levodopa for four weeks, the PSQI score was reduced to a mean of 4.666 ± 2.839 (P-value: 0.0001).

While in the gabapentin group, the mean baseline IRLSS was 26.071 ±7.93, and after treatment for four weeks, IRLSS reduced to 5.357 ± 1.392. The mean baseline PSQI score was 14.857 ± 3.254, and after treatment for four weeks, it was reduced to 2.992 ± 0.916 (P-value: 0.0001) (Table 3).

**Table 3:** Pre- and post-treatment scores of IRLSS and PSQI Abbreviations. Pre-IRLSS: pretreatment international restless leg syndrome score; Post-IRLSS: post-treatment international restless leg syndrome score; Pre-PSQI: pre-treatment Pittsburgh Sleep Quality Index scale; Post-PSQI: pre-treatment Pittsburgh Sleep Quality Index scale

Levodopa-C				Gabapentin			
Parameters	Mean	SD	P-value	Parameters	Mean	SD	P-value
Pre-IRLSS	24.333	5.928	P=0.00001	Pre-IRLSS	26.071	7.936	P=0.00001
Post-IRLSS	8.666	3.312		Post-IRLSS	5.357	1.392	
Pre-PSQI	13.583	3.396	P=0.00001	Pre-PSQI	14.857	3.254	P=0.00001
Post-PSQI	4.666	2.839		Post-PSQI	2.992	0.916	

## Discussion

End-stage renal disease patients on maintenance hemodialysis suffer from multiple complications. Restless leg syndrome is one of them, having a very high prevalence as compared to the general population. It affects not only physical status but is also a major cause of sleep disturbance as well as decreases the quality of life and increases cardiovascular mortality. The exact pathophysiology is still not understood, but the dopaminergic system is supposed to be the major cause of restless leg syndrome, either due to decreased synthesis or decreased number of receptors [10].

American Academy of Sleep Medicine and clinical practice guidelines recommend levodopa as a class A drug for RLS, while the European societies recommend gabapentin as a class A drug. In our study, we diagnosed RLS in 27.7% of patients that were randomly assigned with levodopa-C (46.2%) and gabapentin (53.8%). Both groups were assessed before and after the four weeks of treatment using the IRLSS scale and the Pittsburgh Sleep Quality Index scale.

Dopamine agonists significantly reduce the symptoms of RLS like pain and irritation within a short period of time after intake [11]. But symptoms reoccur after a few hours due to rebounding and augmentation [12]. Our study found that levodopa reduced the symptoms of RLS significantly but was less effective in general health and social functions. Similar findings were also reported in 2004 by Micozkadıoglu et al. [13]. Pre-treatment mean IRLSS in the levodopa group was 24.333 with PSQI mean score of 13.583. After treatment, the scores were reduced to IRLSS of 8.666 and the PSQI mean score of 4.666 with a P-value of 0.0001.

In our study, gabapentin was found to be effective not only in relieving symptoms of RLS but also in improving the quality of life, duration, latency, and reduction in sleep disturbance [14]. Our study found that in the gabapentin group, IRLSS reduced from the pre-treatment mean value of 26.0714 to the post-treatment mean value of 5.357, and the PSQI mean score improved from the pre-treatment value of 14.857 to the post-treatment mean value of 2.99 with a p-value of 0.0001.

Gabapentin has shown superiority in relieving symptoms over levodopa, with the main reduction in IRLSS was 21, while in levodopa, it was 15 [8,13]. In our study, Gabapentin also showed dramatic improvement in the quality of sleep with a PSQI score reduction of 12, and in levodopa, it was around 9; similar findings were reported by Razazian et al. [8] and Micozkadıoglu et al. [13].

## Conclusions

Restless leg syndrome is associated with impaired quality of life and increased cardiovascular mortality as well as anxiety and depression in hemodialysis patients. Levodopa and gabapentin significantly improved the symptoms of RLS. Gabapentin is better in improving the quality of sleep with fewer chances of rebounding and augmentation, as seen with levodopa.
